# Insecticide resistance is mediated by multiple mechanisms in recently introduced *Aedes aegypti* from Madeira Island (Portugal)

**DOI:** 10.1371/journal.pntd.0005799

**Published:** 2017-07-24

**Authors:** Gonçalo Seixas, Linda Grigoraki, David Weetman, José Luís Vicente, Ana Clara Silva, João Pinto, John Vontas, Carla Alexandra Sousa

**Affiliations:** 1 Global Health and Tropical Medicine, Instituto de Higiene e Medicina Tropical, Universidade Nova de Lisboa, Lisboa, Portugal; 2 Institute of Molecular Biology and Biotechnology, Foundation of Research and Technology, Heraklion, Greece; 3 Department of Vector Biology, Liverpool School of Tropical Medicine, Liverpool, United Kingdom; 4 Departamento de Planeamento, Saúde e Administração Geral do Instituto de Administração da Saúde e Assuntos Sociais, IP-RAM, Funchal, Madeira, Portugal; 5 Department of Crop Science, Agricultural University of Athens, Athens, Greece; Centers for Disease Control and Prevention, UNITED STATES

## Abstract

**Background:**

*Aedes aegypti* is a major mosquito vector of arboviruses, including dengue, chikungunya and Zika. In 2005, *Ae*. *aegypti* was identified for the first time in Madeira Island. Despite an initial insecticide-based vector control program, the species expanded throughout the Southern coast of the island, suggesting the presence of insecticide resistance. Here, we characterized the insecticide resistance status and the underlying mechanisms of two populations of *Ae*. *aegypti* from Madeira Island, Funchal and Paúl do Mar.

**Methodology/Principal findings:**

WHO susceptibility bioassays indicated resistance to cyfluthrin, permethrin, fenitrothion and bendiocarb. Use of synergists significantly increased mortality rates, and biochemical assays indicated elevated activities of detoxification enzymes, suggesting the importance of metabolic resistance. Microarray-based transcriptome analysis detected significant upregulation in both populations of nine cytochrome P450 oxidase genes (including four known pyrethroid metabolizing enzymes), the organophosphate metabolizer *CCEae3a*, Glutathione-S-transferases, and multiple putative cuticle proteins. Genotyping of knockdown resistance loci linked to pyrethroid resistance revealed fixation of the 1534C mutation, and presence with moderate frequencies of the V1016I mutation in each population.

**Conclusions/Significance:**

Significant resistance to three major insecticide classes (pyrethroid, carbamate and organophosphate) is present in *Ae*. *aegypti* from Madeira Island, and appears to be mediated by multiple mechanisms. Implementation of appropriate resistance management strategies including rotation of insecticides with alternative modes of action, and methods other than chemical-based vector control are strongly advised to delay or reverse the spread of resistance and achieve efficient control.

## Author summary

*Aedes aegypti* is the major mosquito vector of dengue, chikungunya and Zika worldwide. After its introduction in Madeira, it took a few years for the first dengue outbreak to occur in the region. Control strategies rely mostly on the use of insecticides but their efficiency is often being hampered by the ability of mosquitoes to resist to the compounds used. In fact, previous vector control programs using insecticides failed to eradicate, or even, to limit the spread of *Ae*. *aegypti* in Funchal, and now, the mosquito is widely distributed throughout the southern coast of the island. Bioassays to determine insecticide susceptibility profiles were carried-out in two populations of Madeira Island and the molecular mechanisms underlying the observed insecticide resistance phenotype were investigated. Transcription levels of detoxification genes were analysed, and screenings for *kdr* mutations, V1016I and F1534C, associated with pyrethroid resistance were performed. Our study showed the up-regulation of several detoxification genes of multiple enzyme families associated with metabolic resistance, and the presence of the two *kdr* mutations, with the F1534C being fixed. Another suggested mechanism probably involved in the resistance phenotype is cuticle thickening, as several cuticle genes were found overexpressed. This study reinforces the importance of alternative control strategies to suppress *Ae*. *aegypti* population and thus reduce the likelihood of arbovirus transmission in the region.

## Introduction

*Aedes aegypti* (Linnaeus, 1762) is the most important vector of dengue, chikungunya and Zika viruses. Originally from Africa, this mosquito species invaded other territories and currently has a worldwide tropical distribution, probably aided by globalization [1]. Of major concern is that novel arboviral outbreaks have occurred following the establishment of *Ae*. *aegypti* in new areas [2,3]. In Europe, the most recent example of invasion and subsequent arboviral outbreak is the case of Madeira Island, a Portuguese territory in the Atlantic Ocean. In 2005, *Ae*. *aegypti* was first recorded in the Santa Luzia parish of Funchal (the capital city of Madeira) and soon the vector spread into other parishes of Funchal [4,5]. Control measures based on source reduction were soon implement together with insecticide spraying mainly with pyrethroid insecticides, and application of *Bacillus thuringiensis israelensis* (*Bti*) for larval control [6,7]. This insecticide-based strategy was applied only in Santa Luzia municipality of Funchal, during the years 2006–2008. This strategy included outdoor ULV applications with alfa-cypermethrin and indoor sprayings with tetramethrin and d-fenothrin, in households, and pyrethrins, in warehouses and similar facilities. A liquid formulation of *Bti* was used to treat water fountains or other breeding sites that had standing water [7]. Despite the vector control measures, *Ae*. *aegypti* continued to expand throughout the entire south coast of the island [6].

In 2012, a dengue outbreak was declared by the Madeira Health Authorities with a total of 2,168 cases reported from September 2012 to March 2013 [7]. In this period, a total of 78 imported dengue cases were notified in 13 European countries, consisting of travelers that had visited the island during the outbreak [7]. This event was of particular public health concern given that competent dengue vector *Aedes albopictus* populations are established in southern Europe and have been implicated in autochthonous transmission of dengue and chikungunya viruses in Italy, France and Croatia [8–10].

Insecticide-based vector control efforts did not succeed in reducing the mosquito population, which thrived along the southern coast of the island. This is the most densely inhabited part of the island, resulting in a great availability of human hosts and breeding sites, which coupled with favourable climatic conditions may explain the rapid establishment of *Ae*. *aegypti*. Insecticide resistance might also have contributed to the apparently limited effectiveness of the implemented insecticide-based control measures, but investigation to date has been limited.

Reduced susceptibility to insecticides has been reported frequently in *Ae*. *aegypti* and is primarily associated with two major mechanisms of insecticide resistance, metabolic detoxification and target-site mutations. Over-expression of genes belonging to esterase, Glutathione-S-transferase (GST) and cytochrome P450 oxidase detoxification enzyme families has been reported in insecticide resistant *Ae*. *aegypti* populations from a broad range of geographic locations (reviewed in [11,12]). The role of several detoxification enzymes in insecticide resistance, including six P450s in pyrethroid resistance and the esterase *CCEae3a* in organophosphate resistance has been confirmed by *in vitro* and/or *in vivo* functional validation studies [13,14].

Multiple point mutations have been identified in the voltage-gated sodium channel gene (*Vgsc*) of pyrethroid-resistant *Ae*. *aegypti* populations worldwide. Of these, mutations at two codons are most commonly involved in resistance to pyrethroids in *Ae*. *aegypti*, V1016G or I and F1534C, which may act multiplicatively, especially in combination with an additional mutation, S989P (reviewed in [12]).

Cuticle thickening has also been implicated in insecticide resistance by interfering with the penetration of the insecticide and thus with the amount and rate of insecticide that reaches its target-site [15]. This type of resistance has been documented in several insect species including disease vectors [16–18], and genes encoding cuticle proteins have been found to be over-expressed in insecticide resistance strains of *Ae*. *aegypti* and *Ae*. *albopictus*, as well as *Anopheles stephensi*, and *Culex pipiens pallens* [19–22].

A previous study [23] detected the V1016I and F1534C point mutations in the *Vgsc* gene of *Ae*. *aegypti* from Madeira suggesting that target-site resistance is present on the island. However, no information on the prevalence of insecticide susceptibility, nor on other mechanisms of resistance in Madeira are available to date.

In order to clarify possible causes of inefficacy of insecticide-based control measures implemented on the island, and to assist health authorities in the planning of new interventions, we characterised resistance phenotypes and underlying mechanisms in *Ae*. *aegypti* from Madeira.

## Methods

### Study site and mosquito sampling

The archipelago of Madeira comprises seven islands and two islets situated in the north Atlantic *ca*. 685 km off the coast of Morocco, West Africa. Madeira is the largest island with 742 km^2^ and a population of around 270,000 inhabitants. The island has a mountainous topography and most of the population lives along the coast, especially in the south, where the capital Funchal is located (32°39′4″N 16°54′35″W) and where nearly half of the population lives. This area is densely populated and domestic flower pots, which are the major breeding sites for *Ae*. *aegypti* in the island, are very abundant [24]. The subtropical climate, hilly landscape with exuberant Laurissilva forest and relative proximity to the European continent make this insular territory a popular tourist destination [25].

*Aedes aegypti* were sampled by ovitrap collections carried out in Funchal and Paúl do Mar between September and November 2013. Ovitrap distribution in both localities is available in the citizen science online platform (http://iasaude.sras.gov-madeira.pt/naomosquito/). No specific permits were required for the described field collections. Immatures were reared to adulthood for subsequent use in insecticide susceptibility bioassays. Mosquito rearing and bioassays where performed in the facilities of Direção Regional da Agricultura, provided through a cooperation agreement with the Instituto de Administração da Saúde e Assuntos Sociais (IASAUDE). The *Ae*. *aegypti* Rockefeller strain was used as susceptible reference colony. Mosquitoes were reared in a controlled environment with stable temperature (26±2°C), relative humidity (70±5%) and photoperiod (12h/12h light/dark). A subset of non-insecticide exposed females, which emerged from field collected immatures and from the Rockefeller reference susceptible strain were frozen in liquid nitrogen for subsequent use in biochemical assays. In addition, individuals from the Funchal and Paul do Mar populations, as well as individuals from the susceptible laboratory colonies Rockefeller and New Orleans were stored in RNALater (Invitrogen) to be used in the gene expression analysis.

### Susceptibility bioassays

Bioassays were carried out with 3–5day old non-blood fed females using WHO insecticide susceptibility tests and protocols [26,27]. Filter papers impregnated with insecticide (bendiocarb 0.1%, cyfluthrin 0.15%, fenitrothion 1.0% and permethrin 0.75%) were provided by WHO-University of Sains Malaysia (Penang, Malaysia). Insecticides were chosen according to the three main classes of chemicals allowed to be used in vector control in the region, and based on previous knowledge regarding insecticide resistance status of the *Ae*. *aegypti* populations [7]. Females were exposed to the insecticide in groups of 20–25 per tube, for one hour. Four or five replicates per insecticide were used, depending in mosquito availability. After exposure, mosquitoes were transferred to a holding tube and supplied with a 10% sugar solution on a cotton pad. Mortality was scored 24 hours after exposure. The susceptibility status of each mosquito population was assessed according to WHO recommendations, in which a mosquito population is deemed resistant to a given insecticide if mortality rates are below 90% [26,27] when testing a minimum of 100 specimens. The surviving mosquitoes (considered resistant) and dead (considered susceptible) were stored individually in 1.5ml tubes filled with silica gel desiccant for DNA-based analysis.

In addition, WHO susceptibility tests were carried out with pre-exposure to synergists to block the action of P450s, esterases and GSTs, in order to assess presence of metabolic resistance. Females were exposed to papers impregnated with 4% piperonyl butoxide (PBO) or 8% diethyl maleate (DEM) for one hour and then immediately exposed to each insecticide or to control papers as described above. Mortality rates were scored after 24 hours.

### Biochemical assays

Biochemical assays were performed to quantify the enzymatic activity of the major detoxification families: esterases, Glutathione-S transferases (GST) and cytochrome P450 oxidases (MFO), following WHO protocols [28]. Forty 3–5 days- old individual females from Funchal, Paúl do Mar and an equal number of the Rockefeller reference strain were used in each assay. Comparisons of enzyme activity between field and reference mosquitoes were tested using a Mann-Whitney non-parametric analysis using Graphpad Prism v 6.03.

### Microarray: RNA extraction, labeling and hybridization

Gene expression analysis was carried out at the Liverpool School of Tropical Medicine, UK. Three day-old females, non-blood fed and not exposed to insecticides, that were F1 progeny of mosquitoes collected in Funchal and Paúl do Mar were compared to three day-old non-blood fed, insecticide unexposed females from the susceptible reference colony Rockefeller. In addition, the Funchal population was compared to the second susceptible reference colony New Orleans, to further reduce the possibility that differences observed in expression levels could be related to differences in the genetic background of the laboratory strains unrelated to phenotype. Total RNA was extracted from four replicate batches of five mosquitoes using the Arcturus PicoPure RNA isolation kit (Applied Biosystems). In all cases, RNA was treated with DNAse using the RNase-free DNase Set (Qiagen), according to the manufacturer’s instructions. Quantity and quality of the RNA extracts were evaluated with a Nanodrop spectrophotometer (Nanodrop Technologies) and a 2100 Bioanalyzer (Agilent Technologies), respectively. The RNA pools were amplified and labeled using the Low Input Quick Amp Labeling Kit (Agilent Technologies). Quality and quantity of labeled cRNA was assessed as above before further use. Four hybridizations for each comparison (*i*.*e*. Funchal vs New Orleans, Funchal vs Rockefeller and Paul do Mar vs Rockefeller) were performed using the 15k Agilent “*Aedes* microarray” (ArrayExpress accession number A-MEXP-1966). After 17 hours of hybridization at 65°C, the array was washed to remove non-specifically bound probes, using Agilent microarray washing buffers. Scanning was performed immediately after washing on an Agilent G2205B microarray scanner.

### Microarray data analysis

Data processing was performed using the Agilent Feature extraction software and analysis of normalized data used Genespring v13. A strict filtering criterion was used for inclusion of probes where all had to be detectable (or marginal) in every array across each dataset, resulting in data from 9083 acceptable probes. Probability of differential expression was determined by a one-sample *t*-test (null hypothesis of a ratio of field/colony sample expression of 1) with the *P*-value threshold set at *P*<0.05. A fold change threshold of FC>2, or FC<-2 (for underexpressed probes) was also implemented. We employed a replication criterion for significance, such that a gene was considered differentially expressed if the probability and fold-change thresholds were met for each of the three comparisons with the susceptible reference strains. Although individually the use of a threshold of alpha = 0.05 would lead to a high expected number of false positives (N≈450, ignoring the additional FC criterion), the use of a strict 3/3 replication criterion reduces this dramatically to N≈1 [29], again ignoring additional stringency from the FC criterion. Owing to this strict replication procedure we also identified probes as potentially significant if they exhibited P<0.05 in 2/3 analyses and an extreme level of expression (FC>20). For representation but not assessment of significance, fold changes were averaged and P-values combined using Fisher’s method for combining probabilities.

### Microarray validation by qRT-PCR

The transcription level of candidate overexpressed genes was validated by qRT-PCR in the Funchal population. Two micrograms of DNAse-treated RNA from each sample (four biological replicates for each strain: Funchal, New Orleans and Rockefeller) were reverse-transcribed using oligo(dT)_20_ (Invitrogen) and Superscript III (Invitrogen). Amplification reactions of 25μl final volume were performed in a MiniOpticon Two-Color Real-Time PCR Detection System (BioRad) using 2μl of 1/25 diluted cDNA, 0.2μM primers (S1 Table) and Kapa SYBR FAST qPCR Master Mix (Kapa-Biosystems). For normalization of results, the ribosomal proteins L8_ AAEL000987 and S7 _AAEL009496 were used [30]. A fivefold dilution series of pooled cDNA was used to assess the efficiency of the qPCR reaction for each gene specific primer pair. A no template control (NTC) was included to detect contamination, and a melting curve analysis was done to check for the presence of a unique PCR product. The thermal profile of reactions was 95°C for 3min followed by 40 cycles of 95°C for 15sec, 58°C 30sec and 60°C for 30sec. Relative expression analysis was performed according to Pfaffl [31].

### DNA isolation and *kdr* genotyping

A subsample of mosquitoes phenotyped as susceptible or resistant by WHO assays to pyrethroid insecticides (without pre-exposure to synergists) were genotyped for the presence of the two previously-detected mutations in the *Vgsc* gene [23]. Genomic DNA was extracted according to Collins et al [32]. Two allele-specific PCR assays (AS-PCR) were used to genotype *kdr* mutations V1016I and F1534C [23]. For the V1016I mutation, the protocol used was adapted from Saavedra-Rodriguez et al [33]. Amplifications were carried out in 25 μl of reaction mixture containing 1X buffer, 3 mM of MgCl_2_, 0.2 mM of each dNTP, 0.1 μM of primers Val1016f, Iso1016f and Iso1016r and 1U of *Taq* DNA polymerase. The PCR conditions were identical to those described in Saavedra-Rodriguez et al [33]. PCR products were separated by electrophoresis (90 minutes at 90V) in an ethidium bromide-stained 3% agarose gel and photographed under UV light.

The tetra-primer PCR assay described in Harris et al [34] was used to genotype the F1534C mutation. Each reaction of 25 μl contained 1X PCR buffer, 2.5 mM MgCl_2_, 0.4 mM of each dNTP, 0.25 μM of primers AaEx31P, AaEx31Q, AaEx31wt and AaEx31mut and 1 U of *Taq* DNA polymerase. The cycling conditions were the same used in Harris et al [34]. PCR products were size-fractioned by electrophoresis in ethidium bromide stained 2% agarose gels at 100V (45 minutes) and photographed under UV light.

All PCR assays contained negative controls (*i*.*e*. no DNA template) and positive controls, consisting of samples of known genotype confirmed by DNA sequencing [23].

## Results

### Susceptibility bioassays

**Fig 1:**
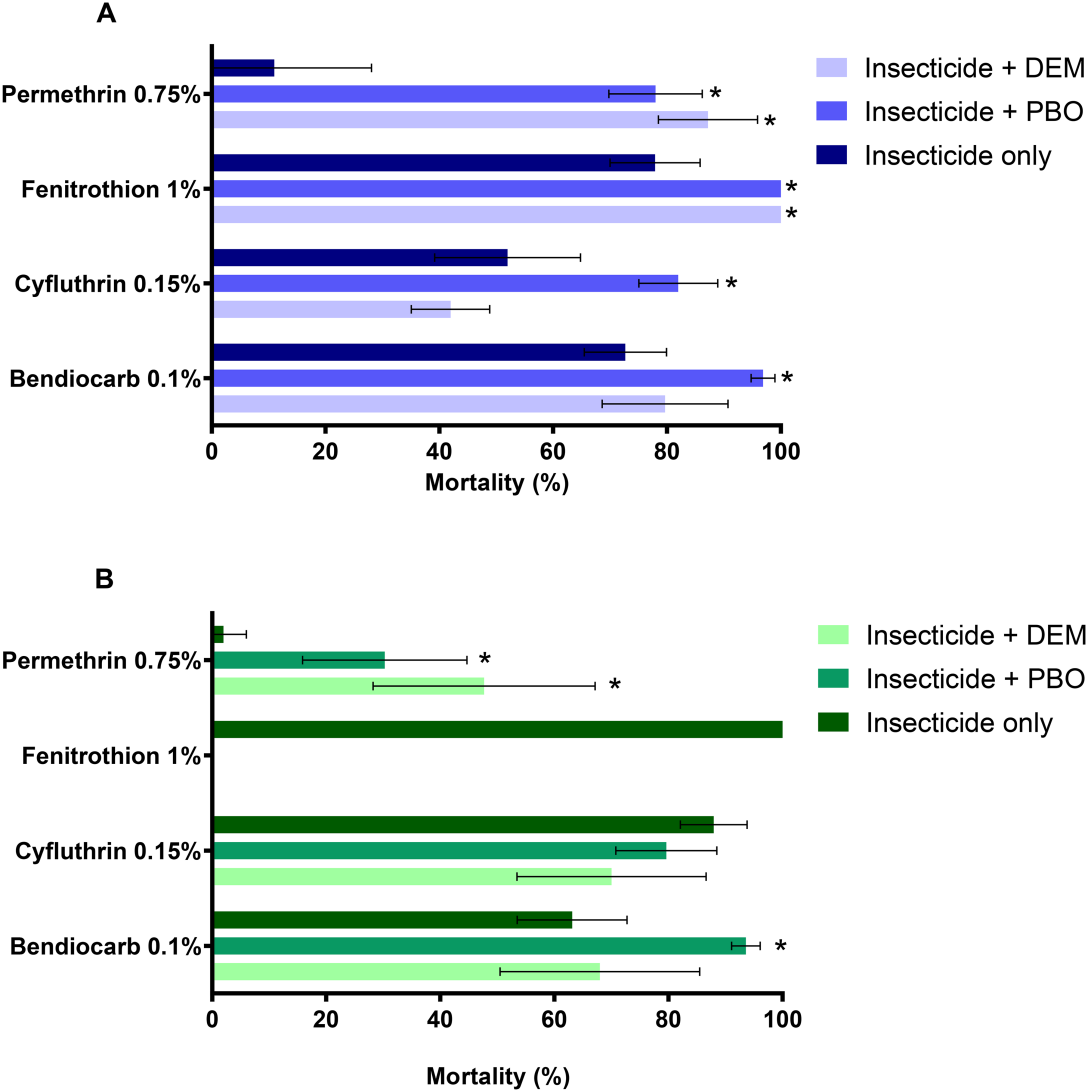
Susceptibility levels of *Ae*. *aegypti* from Funchal (A) and Paúl do Mar (B) to insecticides. * Significant differences in mortality rates between exposures with and without synergists (Fisher’s exact test, *P*< 0.05). The error bars represent standard deviation.

*Aedes aegypti* from Funchal were found to be resistant to all insecticides tested (Fig 1A), with mortality rates ranging between 10.9% (after permethrin exposure) and 77.5% (after fenitrothion exposure). Mortality rates increased significantly when females were exposed to one or both of the synergists before the insecticide, suggesting involvement of metabolic resistance (Fig 1A). This was particularly evident for permethrin, after exposure to either PBO or DEM, and also for fenitrothion, for which complete restoration of susceptibility was attained with both synergists. For cyfluthrin and bendiocarb a significant increase in mortality was observed after exposure with PBO only.

Resistance to pyrethroids and carbamates was also observed in the Paúl do Mar population, with mortality rates between 2% (permethrin), 63.1% (bendiocarb) and 88% (cyfluthrin) (Fig 1B). In contrast, exposure to fenitrothion yielded 100% mortality indicating full susceptibility to this insecticide. As in the Funchal population, synergist assays suggest the presence of metabolic resistance (Fig 1B). Exposure to both synergists, before insecticide contact, led to a significant increase in mortality rates with permethrin. In addition, near-full susceptibility to bendiocarb was achieved when previously exposing these mosquitoes to PBO. Synergist assays with cyfluthrin, in this *Ae*. *aegypti* population, did significantly alter the mortality rate. No mortality was observed in the control mosquitoes whether exposed to control papers alone or to synergists (with no insecticide).

### Biochemical assays

**Fig 2:**
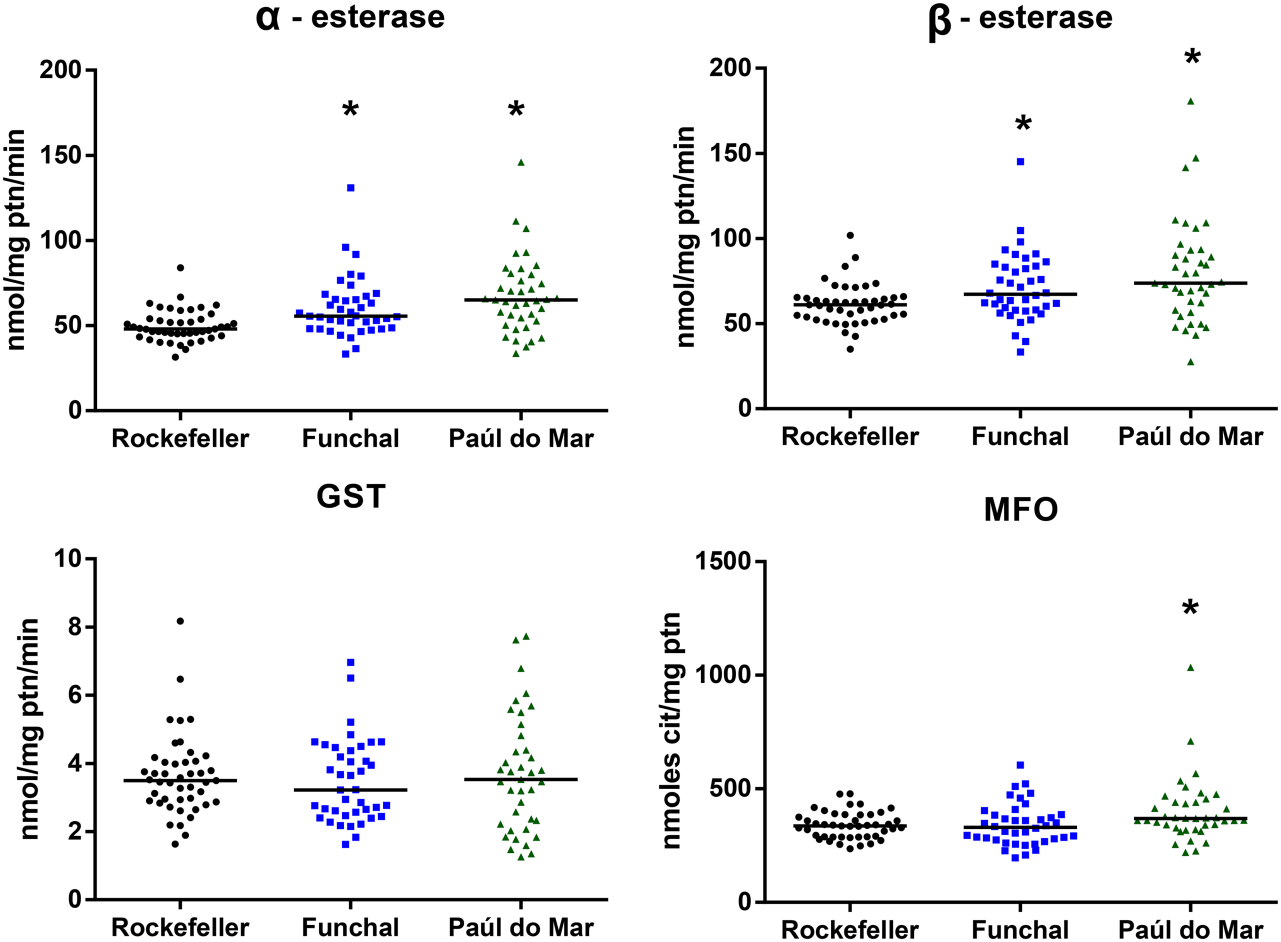
Activity profile of esterases, GST and mixed function oxidases (MFO) enzyme families of *Ae*. *aegypti* from Funchal and Paúl do Mar. * Significant differences in enzymatic activities between the wild population and the Rockefeller susceptible strain (Mann-Whitney test, *P* < 0.05).

A significantly higher enzymatic activity was detected for both α- and β-esterases in both populations when compared to the susceptible Rockefeller reference strain (Mann-Whitney tests, *P*<0.05), while no significant difference was observed in the enzymatic activity of GSTs. A statistically significant difference was seen in mixed function oxidases only in the Paúl do Mar population (*P* = 0.01). Funchal population did not show differences in the enzymatic activity of this enzyme family (*P* = 0.7204) (Fig 2).

### Microarray analysis of differential expression

**Fig 3:**
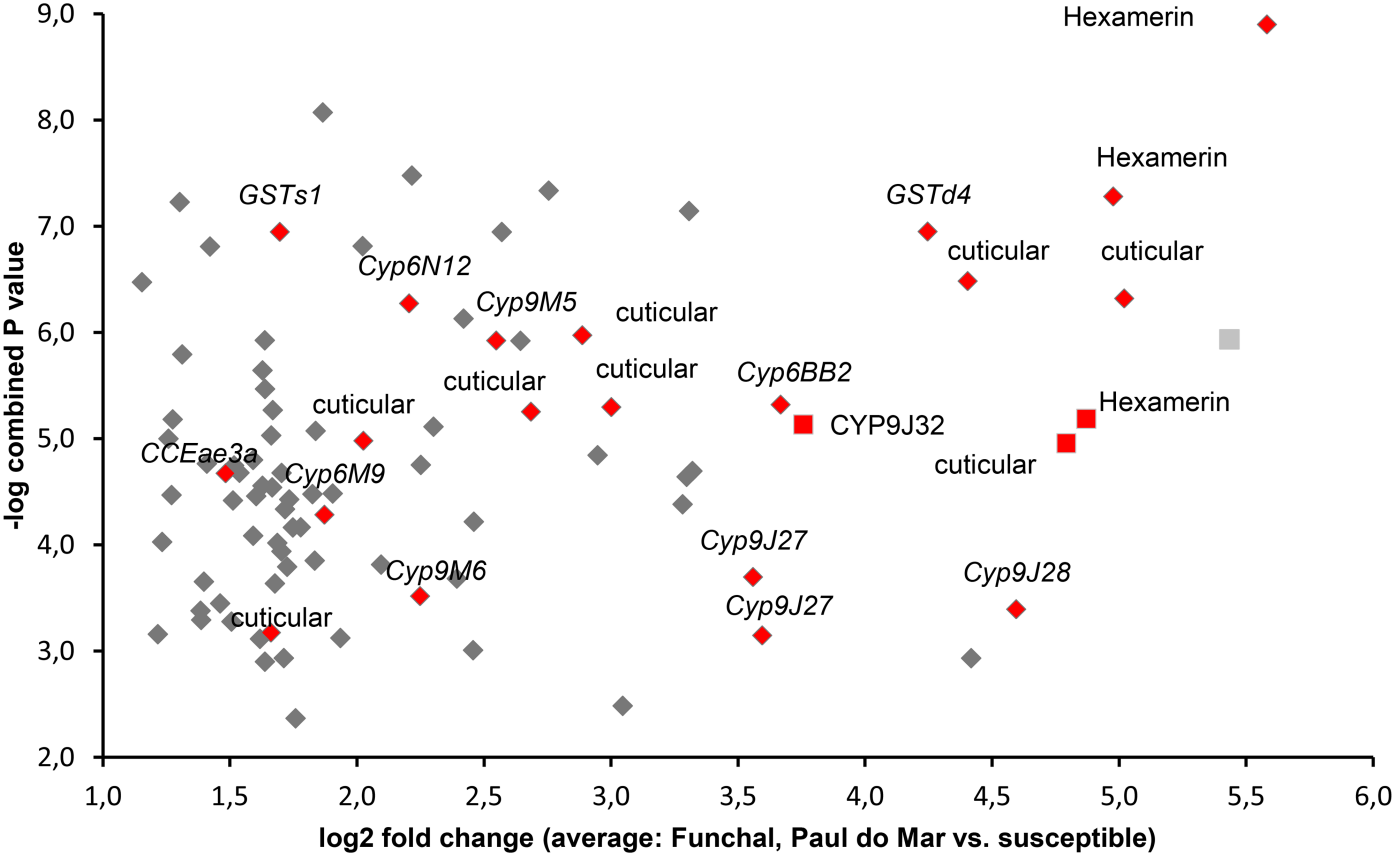
Commonly up-regulated transcripts in *Ae*. *aegypti* populations from Funchal and Paúl do Mar. A rhombus shape is used for transcripts meeting the criteria Fold Change >2 and P<0.05 in all three comparisons performed. Among these genes are the rer1 protein (possibly involved in the retrieval of endoplasmic reticulum proteins), a lyposomal aspartic protease precursor and many genes with unknown function. A square shape is used for potentially significant up-regulated transcripts, exhibiting a P<0.05 in 2/3 analyses and an extreme level of expression (FC>20). All transcripts falling into the categories of detoxification genes, hexamerins or genes encoding for cuticular proteins are shown with red color. *Cyp9J27* is present in two distinct locations in the genome and so is represented twice, though with different accession numbers (Table 1).

From a total of 9083 probes passing quality control, 141 were differentially expressed (|Fold Change|>2, *P*<0.05, for three out of three comparisons to susceptible strains) across the Funchal and Paul do Mar populations (S1 Table). Among the 86 probes which were up-regulated (Fig 3), 11 genes were members of the three detoxification enzyme super-families (P450s, GSTs and Carboxyl/choline esterases) (Table 1). A further gene, *Cyp9J32* was also included in the table with the over-expressed genes as it showed extreme over-expression, and only marginally missed the threshold P-value in one (out of three) comparison. Thus we considered this as a false negative, resulting from our strict filtering procedure (Table 1). The P450 oxidases had the highest representation with nine genes, all from the CYP6 and CYP9 sub-families and included four known pyrethroid metabolizers, of which *Cyp9J32* and *Cyp9J28* were particularly strongly over-expressed (FC>20). Other detoxification genes found overexpressed in both wild populations were the *GSTd4*, *GSTd1* and *CCEae3a*, which metabolizes temephos-oxon (the toxic form of the larvicide temephos) and thus may be of relevance for resistance in the populations, although we did not evaluate temephos resistance in this study.

**Table 1:** Commonly overexpressed transcripts in *Ae*. *aegypti* from Funchal and Paul do Mar belonging to detoxification gene families.

Class of detoxification gene	Gene accession number	Gene name	Funchal vs Rockefeller (FC)	*P*-value	Funchal vs New Orleans (FC)	P-value	Paul do Mar vs Rockefeller (FC)	P-value
P450s	AAEL008846	***Cyp9J32***	41	0.006	53	0.004	10	0.060
	AAEL014617	***Cyp9J28***	31	0.028	30	0.019	10	0.008
	AAEL014893	***Cyp6BB2***	15	0.005	19	7*10^−4^	3.6	0.006
	AAEL014607	*Cyp9J27*	14	0.021	18	0.018	3.5	0.023
	AAEL014616	*Cyp9J27*	14	0.020	16	0.016	4.6	0.006
	AAEL001288	*Cyp9M5*	6.6	6*10^−4^	8.5	0.009	2.3	0.001
	AAEL001312	***Cyp9M6***	5.4	0.015	4.7	0.006	4	0.031
	AAEL009124	*Cyp6N12*	5.1	3*10^−4^	6.3	0.003	2.2	0.002
	AAEL017297	*Cyp6M9*	4.2	0.007	3.7	0.001	3	0.047
Esterases	AAEL005112	***CCEae3a***	2.7	0.009	3.4	0.012	2.1	0.001
GSTs	AAEL001054	*GSTd4*	24	4*10^−4^	22	2*10^−4^	9.8	0.004
	AAEL011741	*GSTs1*	4.9	2*10^−4^	2.5	7*10^−5^	2.2	0.032

FC represents the relative fold change in expression in the Funchal or Paul do Mar population compared to the respective susceptible colony. Bold type indicates a known insecticide metabolizer [13,14,40].

Beyond genes belonging to detoxification gene families the analysis revealed also the overexpression of eight transcripts encoding putative cuticle proteins, which have been implicated in resistance through lower insecticide penetration and also of three transcripts (AAEL011169, AAEL013759 and AAEL000765, which was highly and significantly, P<0.05, up-regulated in 2/3 comparisons) encoding hexamerins, which are involved in cellular trafficking and have previously been linked to insecticide resistance [35] (Fig 3, S1 Table).

### qRT-PCR validation

Quantitative real time PCR was used to validate the differential expression of five candidate genes detected as significantly up-regulated in the Funchal population (for which microarray data *vs*. both susceptible colonies was available) compared to the two susceptible colonies. We tested the highly overexpressed P450s *Cyp9J32* and *Cyp9J28*, the more highly overexpressed of the glutathione S transferases, *GSTd4*, the more highly overexpressed of the hexamerins, AAEL013757 and the more highly overexpressed member of genes encoding for putative cuticular proteins, AAEL002246 (S1 Table) (Table 2). Although the relative levels of overexpression estimated by qRT-PCR did not correspond closely with values obtained from the microarray experiment, the estimates from qRT-PCR confirmed up-regulation of the tested genes, thus providing validation of the significance indicated by microarrays.

**Table 2:** Validation of the transcriptional up-regulation of five candidate genes through qRT-PCR.

		qRT-PCR	Microarray
Transcript	Reference strain	Fold change (95% CI)	Fold Change
**Hexamerin (AAEL013757)**	Rockefeller	63 (36–90)	57
	New Orleans	123 (54–191)	97
***Cyp9J32* (AAEL008846)**	Rockefeller	17 (13–21)	41
	New Orleans	107 (81–132)	53
**Cuticular (AAEL002246)**	Rockefeller	42 (16–69)	36
	New Orleans	65 (25–105)	51
***Cyp9J28* (AAEL014617)**	Rockefeller	13 (6–21)	31
	New Orleans	104 (63–144)	30
***GSTd4* (AAEL001054)**	Rockefeller	169 (34–305)	24
	New Orleans	268 (148–387)	22

The relative expression ratio of five candidate genes in the Funchal population compared to two susceptible laboratory colonies (New Orleans and Rockefeller) is shown. Values are estimated from four biological replicates and 95% confidence intervals are shown. Estimated values from the microarray experiment are given for comparison.

### *Kdr* genotyping

Genotyping of the *kdr* locus was performed on a total of 91 Funchal and 80 Paúl do Mar specimens that had been previously exposed to cyfluthrin or permethrin (Table 3). The 1534C mutation was found in every specimen genotyped suggesting it may be fixed in both populations, while the V1016I mutation showed moderate and similar frequencies ranging from 17% in Funchal to 23% in Paúl do Mar (Fisher’s exact tests, P = 0.263). Owing to ubiquitous occurrence of the 1534C mutation testing association with resistance was not possible. Although the V1016I frequency was slightly higher in resistant mosquitoes when compared to susceptible ones, there was no significant association between *kdr* genotypes and the resistance phenotype for either insecticide (Fisher’s exact tests, Funchal—cyfluthrin: *P* = 0.491; permethrin: *P* = 0.699; Paúl do Mar—cyfluthrin: *P* = 0.316; permethrin: *P* = 0.219).

**Table 3:** Summary of *kdr* genotyping data in Funchal and Paúl do Mar *Ae*. *aegypti* populations.

			V1016I				F1534C			
Locality	Insecticide	N	V/V	V/I	I/I	F.(I)	F/F	F/C	C/C	F.(C)
**Funchal**	Cyfluthrin resistant	32	20	10	2	0.22	0	0	32	1.00
	Cyfluthrin susceptible	19	15	4	0	0.11	0	0	19	1.00
	Permethrin resistant	32	21	11	0	0.17	0	0	32	1.00
	Permethrin susceptible	8	6	2	0	0.13	0	0	8	1.00
	**Total**	**91**	**62**	**27**	**2**	**0.17**	**0**	**0**	**91**	**1.00**
**Paúl do Mar**	Cyfluthrin resistant	12	6	5	1	0.29	0	0	12	1.00
	Cyfluthrin susceptible	36	21	15	0	0.21	0	0	36	1.00
	Permethrin resistant	30	18	11	1	0.22	0	0	30	1.00
	Permethrin susceptible	2	0	2	0	0.5	0	0	2	1.00
	**Total**	**80**	**45**	**33**	**2**	**0.23**	**0**	**0**	**80**	**1.00**

N: sample size. Values correspond to absolute numbers for each genotype. F.(I) and F.(C) are the relative frequencies of the mutant allele for each mutation analyzed.

## Discussion

The results of this study showed that *Ae*. *aegypti* from Madeira Island is resistant to insecticides of different chemical classes: carbamates (bendiocarb), organophosphates (fenitrothion) and both type I (permethrin) and type II (cyfluthrin) pyrethroids. Diagnostic exposures to these insecticides yielded mortality rates below the thresholds recommended by WHO to consider a mosquito population resistant [26,27], excepting the case of fenitrothion in Paúl do Mar. Combined bioassays with synergists and analysis of detoxification enzyme activities indicated the presence of enzyme-mediated metabolic resistance, and/or cuticular resistance. Pre-exposure to PBO, which inhibits P450s, some esterases and may also enhance cuticular penetration by the insecticide [36] resulted in a significant mortality increase for all insecticides tested. Synergist pre-exposures suggest involvement of the three major detoxification enzyme families in the resistance phenotype of *Ae*. *aegypti* from Madeira island. Biochemical assays only partially agreed with the results obtained by the bioassays with synergists, as significantly elevated enzymatic levels were detected for esterases only. These discrepancies were not completely unexpected as these assays cannot be considered reciprocal. While synergists act as inhibitors of enzymes suspected to be implicated in resistance, biochemical assays are a measure of enzyme activity without a direct link with the resistance phenotype. Furthermore, biochemical assays employ generic substrates which may not be recognized by all variants of these large enzyme families, resulting in reduced sensitivity and specificity [36, 37, 38]. The microarray-based transcriptomic analysis showed overexpression of genes belonging to the three major detoxification enzyme families in agreement with the bioassays with synergists. The majority of overexpressed detoxification genes were cytochrome P450 oxidases, including *Cyp9J32*, *Cyp9J28*, *Cyp9J27*, *Cyp6BB2* and *Cyp9M6*, which have been found overexpressed in pyrethroid resistant *Ae*. *aegypti* populations from multiple countries [12,36,39]. In particular, *Cyp9J28* is an efficient pyrethroid metabolizer [13] that has also been shown to confer reduced susceptibility to deltamethrin when ectopically expressed in *Drosophila melanogaster* [40]. *Cyp9J32*, the most prominent pyrethroid metabolizer in *Ae*. *aegypti*, showing a very high catalytic efficiency against pyrethroids [13], was the most overexpressed P450 gene. The carboxyl-esterase *CCEae3A*, which has previously been associated with resistance to the organophosphate temephos-oxon [14] in both *Ae*. *aegypti* [30] and *Ae*. *albopictus* [41], and acts via sequestration and metabolism of temephos [14], was also among the overexpressed genes in Funchal and Paúl do Mar. This should be taken into account if temephos is considered for vector control in Madeira. The overexpression of two GSTs (*GSTd4* and *GSTs1*) was also revealed by the microarray analysis. Of these, *GSTd4* has been detected as highly overexpressed in the strongly permethrin resistant Singapore SP strain [42].

In addition to detoxification gene families, eight transcripts encoding putative cuticular proteins were up-regulated possibly indicating alteration of the cuticle as a mechanism of resistance. Resistance related to the cuticle by lowering the amount or rate of insecticide penetrating into the body has been reported in insects such as *Helicoverpa armigera* [43], *Drosophila melanogaster* [44], the *Trypanosoma cruzi* vector *Triatoma infestans* [16], and the *Plasmodium* vector *Anopheles funestus* [17]. Thickening of the whole cuticle, as well as the epicuticle layer, due to an increased number of cuticular hydrocarbons, has also been recently described in a multi-resistant strain of the malaria vector *Anopheles gambiae* [18]. Furthermore, there were also three transcripts (one marginally non-significant) encoding hexamerins among the most highly overexpressed genes. The link of hexamerins to insecticide resistance is poorly understood, but previous studies suggest a role for these storage proteins in cuticle formation [45,46]. It is also plausible that composition and thickening of the cuticle might reflect adaptive responses to environmental challenges and/or seasonality, rather than insecticide resistance. Seasonal cuticular variations, mostly associated with adaptation to aridity (desiccation tolerance), have been previously observed in other insect species, including scorpions [47], crickets [48] and more recently in the malaria vector *A*. *coluzzii* [49]. There is evidence that *Ae*. *aegypti* of Madeira derives from a tropical south American source population [23], thus, adaptation of this mosquito to the more temperate climate of Madeira might have involved changes in cuticle composition and thickening.

Genotyping of the *kdr* locus confirmed not only the presence, but probable fixation of the pyrethroid resistance mutation F1534C, in line with previous studies [23]. However, the V1016I pyrethroid resistant mutation showed a significant frequency increase (8% in 2009 [23], to 17% in 2013, this study; Fisher’s exact tests, *P* = 0.019). The role of V1016I in resistance to pyrethroids is currently unclear but frequencies of both mutations appear informative and should be routinely monitored [50]. Hu et al [51] found that the F1534C mutation is more effective in reducing sensitivity of the sodium channel to type I than to type II pyrethroids. This could explain the higher resistance level of *Ae*. *aegypti* from Funchal to permethrin when compared to cyfluthrin. However, reversal of resistance to permethrin with the synergist PBO was comparable to that obtained for cyfluthrin suggesting that resistance to both type I and type II pyrethroids may be primarily mediated by the metabolic activity of cytochrome P450 oxidases. These findings should be taken into consideration by the Health Authorities in Madeira when deciding between type I or type II pyrethroids for vector control. If this chemical class remains an option for chemical control of *Ae*. *aegypti* in Madeira island, the use of type II pyrethroids in combination with the synergist PBO appears to be a more effective option.

### Conclusion

Insecticide resistance mediated by multiple mechanisms was identified in *Ae*. *aegypti* from two localities in Madeira. In addition to target-site (*kdr*) and metabolic resistance, a third mechanism consisting of cuticle thickening may also be involved, confirming that the insecticide resistance phenotype is multifactorial, and consequently is likely to be challenging to reverse. The recent presence of this species in the island and the absence of a continuous, island-wide, insecticide-based control suggests that at least some, and maybe most, of the insecticide resistance mechanisms detected were already present in the colonizing specimens. Thus, the resistance status of these mosquitoes may have played some role in the establishment of this vector in the island, despite the 2006–2008 insecticide-based vector control campaign. With the current knowledge regarding insecticide resistance status and identification of underlying mechanisms, resistance management strategies including mode of action rotation (such as biocides and insect growth regulators), as well as alternative to chemical-based vector control interventions (ranging from environmental management to new paradigms and biotechnology-based approaches) is strongly advised, to control *Ae*. *aegypti* and thus decrease the probability of arbovirus transmission.

## Supporting information

S1 Table List of probes analysed after the quality control and primers used in qPCR. (XLS)
